# The Formation of Polyvinylidene Fluoride Membranes with Tailored Properties via Vapour/Non-Solvent Induced Phase Separation

**DOI:** 10.3390/membranes8030071

**Published:** 2018-09-01

**Authors:** Tiziana Marino, Francesca Russo, Alberto Figoli

**Affiliations:** Institute on Membrane Technology (ITM-CNR), Via Pietro Bucci, Cubo 17C, 870 36 Rende (CS), Italy; t.marino@itm.cnr.it (T.M.); f.russo@itm.cnr.it (F.R.)

**Keywords:** non-toxic triethyl phosphate, membrane preparation, TEP, PVDF membranes, VIPS-NIPS

## Abstract

The present investigation reports as it is possible to prepared polyvinylidene fluoride (PVDF) membranes for microfiltration (MF) and ultrafiltration (UF) applications, by using triethyl phosphate (TEP) as non–toxic solvent in accordance with the Green Chemistry. Casting solutions containing different concentrations of polyethylene glycol (PEG) were prepared in order to study its effect on the final membrane morphology and properties. The possibility to finely modulate membrane properties was also investigated by applying two different membrane preparation techniques, the Non-Solvent Induced Phase Separation (NIPS) and its coupling with Vapour Induced Phase Separation (VIPS). Membranes’ morphology was detected by Scanning Electron Microscopy (SEM). Thickness, porosity, contact angle, pore size and water permeability were also recorded. Both the PEG content in the dope solution and the selected time intervals during which the nascent films were exposed to established relative humidity and temperature were found to play a crucial role in membrane formation. In particular, it was demonstrated as, by varying PEG content between 10 and 20 wt %, and by setting the exposure time to humidity at 0/2.5/5/7.5 min, membranes with different pore diameter and bicontinuous structure, suitable for UF and MF applications, could be easily obtained.

## 1. Introduction

The fifth principle of Green Chemistry, connected with the use of safer solvents and auxiliaries, states that “The use of auxiliary substances (e.g., solvents, separation agents, etc.) should be made unnecessary wherever possible and innocuous when used” [1]. These materials include any compound that does not affect directly the chemical structure of the final product(s) but which is needed for carrying out the desired process. In membrane preparation via phase inversion, solvents represent the main auxiliary material, being used for dissolving the selected polymer and for allowing the phase inversion [2]. Organic diluents, such as *N*,*N*-dimethyl formamide (DMF), *N*,*N*-dimethyl acetamide (DMA) and *N*-methyl pyrrolidone (NMP), although widely used in membrane preparation because of their excellent ability to dissolve polymeric materials, including polyvinylidene fluoride (PVDF), may pose serious health and environment risks. The Sigma Aldrich chemical company [3], according to the Regulation (EC) No 1272/2008 [4], classifies these compounds as highly harmful, as it can be observed from the Material Safety Data Sheet. As reported in Table 1, DMF, DMA and NMP are detrimental, may damage fertility and unborn children (Hazard statement code: H360FD). Therefore, the replacement of highly toxic diluents with less/non-toxic alternatives represents a challenging goal for human and environment protection [5,6].

**Figure 1:**
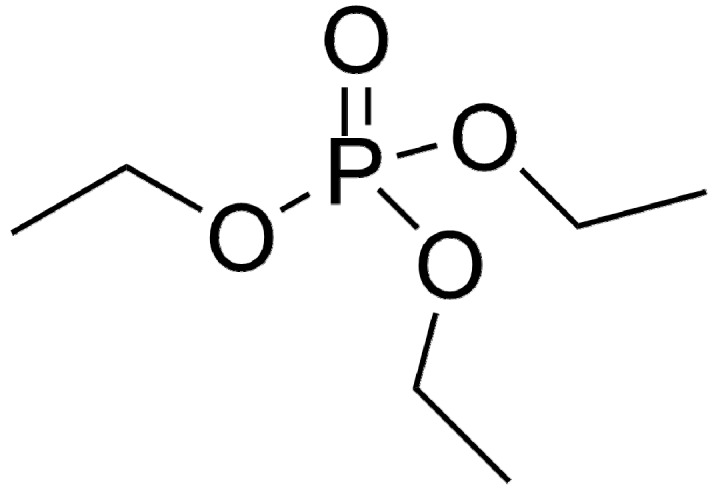
Molecular structure of TEP.

In this context, triethyl phosphate (TEP) (Figure 1) offers the possibility to efficiently prepare polymeric membranes without exposing workers to serious risks for health [6]. 

This solvent is not carcinogenic, teratogenic or mutagenic as the commonly used solvents, and, as stated in its Material Safety Data Sheet, “contains no components considered to be either persistent, bioaccumulative and toxic, or very persistent and very bioaccumulative at levels of 0.1% or higher” (Table 1) [3,7]. The Organisation for Economic Co-operation and Development reports that the bioaccumulation potential and the concern for risk to humans and environment of this solvent are low [8].

The possibility to efficiently substitute the hazards’ solvents with TEP for polymeric membranes production is also confirmed by the growing number of scientific papers [9,10,11,12,13,14,15,16,17,18,19].

Particularly interesting are the TEP ability to dissolve PVDF, which represents one of the most attractive materials for membrane fabrication [20], its high boiling point (215 °C) and its complete miscibility with water and alcohols, widely used as non-solvents during the membrane preparation procedure. PVDF is characterised by outstanding thermal and chemical resistance, and PVDF membranes have been applied for several separation processes [20]. TEP ability to dissolve PVDF is defined by Hansen solubility parameters [12], which denote the distance between PVDF and the solvent in a three-dimensional δd, δp, and δh Hansen space. The lower the δSP (Solvent polymer), δPVDF–S value, the better PVDF is dissolved in the corresponding solvent. TEP solubility parameters are close to organic solvents reported in Table 2, and similar to PVDF, confirming that PVDF-TEP solutions are thermodynamically favored.

Moreover, phase inversion behaviour reported by Chang et al. [11] indicated by the ternary phase diagram of PVDF/TEP/water demonstrated as PVDF/TEP system needs a less water content to promote phase inversion than PVDF/NMP, i.e., TEP is a weaker solvent for the polymer in comparison to the traditional hazard one. Similar results were reported by Bottino et al. [23]. Tao et al. [10] investigated the effects of four solvents including TEP, hexamethylphosphoramide (HMPA), trimethylphosphate (TMP), and DMF on the fabrication of PVDF membranes. It was observed as PVDF membrane polymorphism, thus the polymer crystalline phase, was highly dependent on the solvent dissolving capability. Poorly dissolved solution, obtained with HMPA, had the tendency to form β-phase, while α-phase dominated when PVDF was well dissolved in TEP. Membranes prepared with TMP and DMF exhibited a mixture of α- and β-PVDF phases. Characterization tests showed as membrane prepared with TEP exhibited the highest pure water flux and the steady flux still reaches up to 1860 L/m^2^·h, suggesting that this solvent membrane could be used for producing PVDF micro-filtration membranes. PVDF hollow fiber membranes were produced by Zhang et al. [24] adding TEP to the cooling water bath. By using 40% *w*/*w* TEP in water, hollow fiber become porous symmetric, and manifested high permeability (540 L/m^2^·h). Recently, Chang et al. [11] proposed the use of TEP as a green alternative to NMP for the fabrication of PVDF hollow fiber membranes. It was observed that TEP led to more porous membrane; the porosity reached 92.7% when TEP was used not only as solvent, but also mixed with water, as coagulation medium. Similarly, Lin et al. [25] investigated the influence of TEP as solvent and the precipitation bath composition on the final PVDF membrane morphology. TEP promoted polymeric crystallization, even when a harsh bath (e.g., water) was used. In fact, membrane structure was composed of large spherulites, typical of crystallization phenomenon. TEP/water mixture led to a very uniform membrane matrix, in which stick-like crystallites interlocked into a bi-continuous structure. Liu et al. [14] described the fabrication of PVDF flat sheet membranes with inter-connected pores via a non-solvent assisted thermally induced phase separation process. The metastable polymeric solution was obtained by using TEP as the latent solvent. By acting on both the casting solution and the quench bath composition, asymmetric ultrafiltration (UF) and symmetric microfiltration (MF) membranes were respectively obtained. The proposed Nat-ips method allowed for producing PVDF membranes with inter-connected pores and unimodal pore size distribution. The MF membrane presented a pore size of 0.26 μm and a pure water flux of 1860 L/m^2^·h·bar, while the UF membrane showed a pore size of 0.11 μm and a pure water flux of 120 L/m^2^·h·bar. Registered bovine serum albumin rejection was 97% and 12% for the MF and UF membranes, respectively.

Another attracting membrane preparation procedure is represented by the coupled NIPS-VIPS techniques [26]. In this technique, a preliminary step, during which the nascent films are first exposed to fixed humid air, precedes the immersion in the coagulation bath. Generally, for these systems, the non-solvent is represented by water, thus the water vapour transfer promotes the phase separation. The moist air plays a key role for the polymer precipitation, which is then completed with the immersion in the non-solvent bath. By accurately adjusting the relative humidity and temperature of the air, and, by varying the exposure time, a selected casting solution can be used to fabricate membranes with different morphologies and properties.

This work focuses on the PVDF porous membrane preparation by combining NIPS and VIPS. By carefully controlling the relative humidity and the temperature casting the solution in a climatic chamber, and by exposing the nascent membranes to humidity for different times, it was possible to tailor the membrane morphology and performance. Both PVDF UF and MF membranes with a bicontinuous structure, having different pore size and PWP, were prepared by using TEP as a less toxic solvent. The effect of PEG concentration on the final membrane was also examined.

## 2. Materials and Methods

### 2.1. Chemicals

PVDF (Solef^®^ 6010; Mw = 322 kg/mol) was kindly supplied by Solvay Specialty Polymers (Bollate, Italy). TEP (Sigma Aldrich, Milan, Italy) was used as a solvent without further purification. Polyvinylpyrrolidone (PVP K17, BASF, Ludwigshafen, Germany, Mw = ~9 kg/mol) and PEG (PEG-200, Sigma Aldrich, Milan, Italy, Mw = 200 g/mol) were selected as pore former additives. PVP was desiccated under vacuum at 50 °C for 12 h before use. Bi-distilled water at 15 °C was used as coagulation medium.

### 2.2. Membrane Preparation

In addition, 15 wt % PVDF was dissolved in TEP at 100 °C. When additives were used, 5 wt % PVP and the correct amount of PEG (ranging from 0 to 20 wt %) were added to the polymer–solvent mixture. The solution was maintained under stirring at 100 °C until a homogeneous clear solution was formed. Dope solution was cast on a glass plate by means of a manual applicator equipped with a with reservoir (Elcometer 3700/1 Doctor Blade, Aalen, Germany; adjustable gap size: 30–4000 μm) and set at 250 μm. The casting step was carried out in a climatic chamber (DeltaE srl, Rende, Italy), which allowed to finely control relative humidity and temperature. Specifically, relative humidity and temperature were fixed at 55% and 25 °C, respectively. Membranes were formed via NIPS (immediate polymer precipitation in the water bath) or coupling NIPS with VIPS, i.e., exposing the forming membranes to humid air for a 2.5/5/7.5 min before immersion in the coagulation medium.

The prepared membranes are listed in Table 3. The obtained flat membranes were washed with water at 50 °C for three consecutive times, dried in the open air for 12 h and kept in an oven at 40 °C for 12 h before characterization.

### 2.3. Membrane Characterization

#### 2.3.1. Thickness

A Carl Mahr digital micrometer (Esslingen, Germany, precision of ±0.001 mm) was used to detect Membrane thickness. For each membrane, after measuring the thickness for seven regions, the average value was determined.

#### 2.3.2. Porosity

Porosity was acquired by applying the following Equation (1):

(1) $$\;\mathrm{Porosity}\;(\%)\;=\frac{\frac{{\mathrm{wt}}_{w}-{\;\mathrm{wt}}_{d}}{\rho_{k}}}{\frac{{\mathrm{wt}}_{w}-{\;\mathrm{wt}}_{d}}{\rho_{k}}+\frac{{\mathrm{wt}}_{d}}{\rho_{p}}}\times 100$$

where wt_w_ is the membrane wet weight, wt_d_ is the membrane dry weight, ρ_k_ is the kerosene density and ρ_p_ is the polymer density.

For porosity calculation, three pieces of the same membrane were weighted before and after the immersion in kerosene for 24 h.

#### 2.3.3. Pore Size

CFP-1500 AEXL (Porous Materials Inc., Ithaca, NY, USA) capillary flow porometer instrument was employed for determining membrane pore diameter through the liquid–gas displacement process. Porewick^®^, having a surface tension of 16 dyne/cm, was used as wetting liquid which filled membrane pores and nitrogen as inert gas. Pore size tests were carried out after keeping membranes in Porewick^®^ for 24 h. By gradually increasing nitrogen pressure, the wetting liquid was pushed out from membrane pores. Both gas pressure and permeation flow rate across the membrane were detected. Mean flow pore diameter (d) was determined by applying the following equation [27]:

d = C γ/p,(2)

where C is a constant (0.0286 bar), p is differential gas pressure at which wet flow was one-half the dry flow and γ is the wetting liquid surface tension.

#### 2.3.4. Pure Water Permeability (PWP)

A laboratory cross-flow cell (DeltaE srl, Milan, Italy), operating at 25 °C, was used to carry out PWP experiments. Pure water was forced to pass across the membrane having an area of 8 cm^2^, by means of a gear pump (Tuthill Pump Co., Concord, CA, USA). Before starting the permeability tests, an equilibration period of 30 min was performed (transmembrane pressure was in this case equal to 2 bar). In addition, 1.0/0.8/0.6 bar as transmembrane pressures, separated by a stabilization period of one to another of 20 min, were applied and the permeate was collected in 60 s. PWP was calculated by applying the following Equation (3):

PWP = Q/A t p,(3)

where A is the membrane area (expressed in m^2^); p is the pressure (expressed in bar); Q is the permeate volume in liters; and t is the time (expressed in hours).

#### 2.3.5. Contact Angle

Static contact angle was assessed by using an optical tensiometer (CAM100 Instrument, Nordtest srl, GI, Serravalle Scrivia (AL), Italy) adopting the sessile drop method. For each membrane, five measurements were taken, allowing for calculating the average and the standard deviation.

#### 2.3.6. Scanning Electron Microscopy (SEM)

Zeiss-EVO MA10 microscopy (Carl Zeiss, Oberkochen, Germany) was used to capture SEM pictures of the membranes. In order to observe the cross sectional morphology, membranes were firstly freeze fractured in liquid nitrogen. and then covered, as the membrane surface, with a thin conductive gold layer (Quorum Q150 RS).

## 3. Results and Discussion

### 3.1. Membrane Morphology

**Figure 2:**
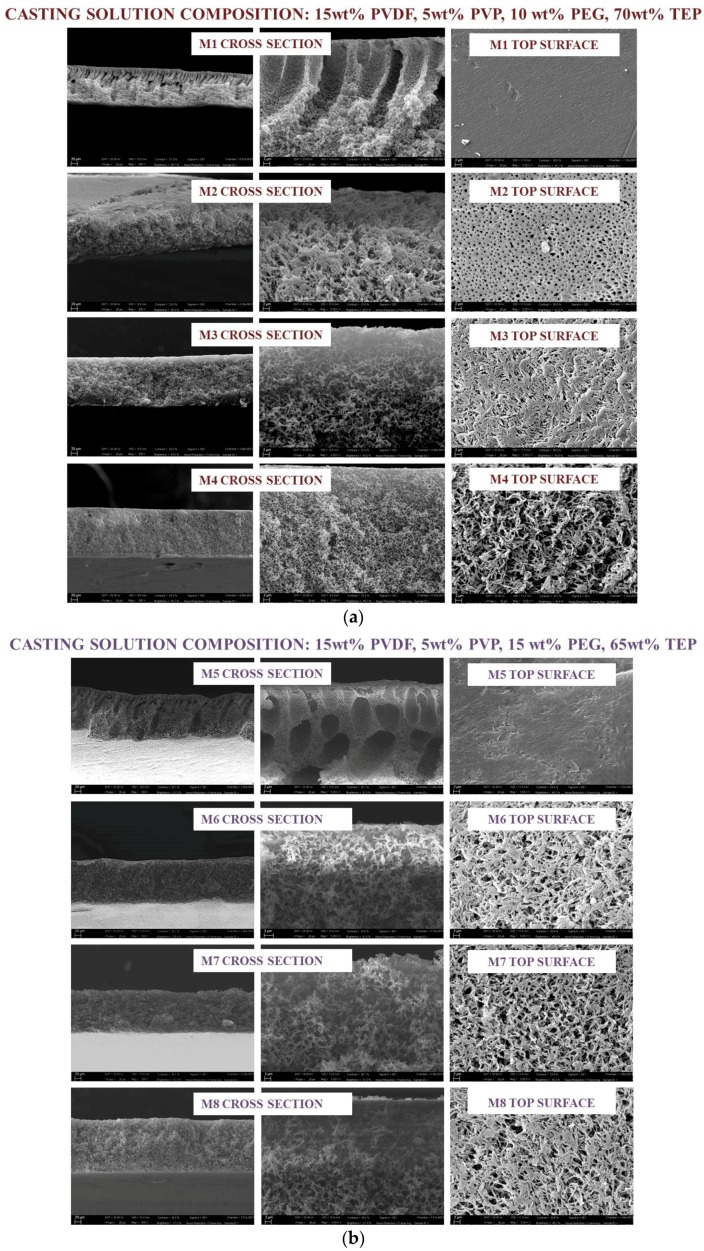

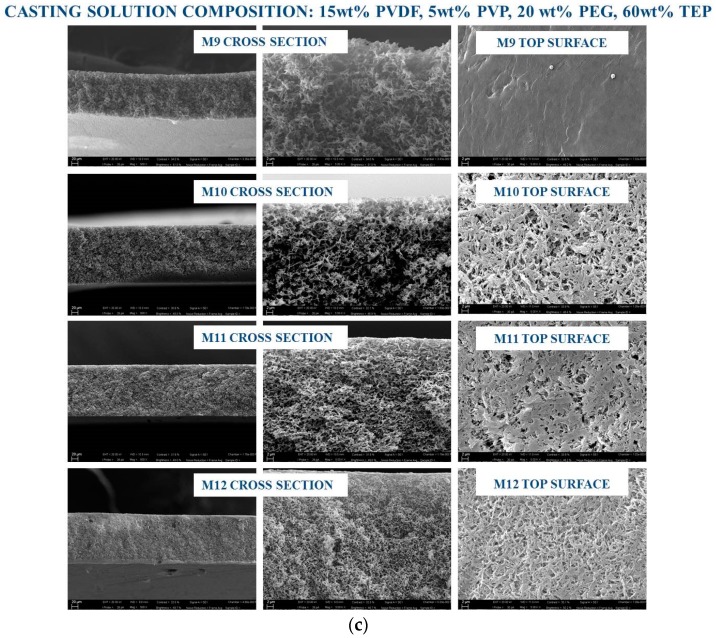
(**a**) Casting solution composition 15 wt % PVDF, 5 wt % PVP, 10 wt % PEG, 70 wt % TEP; (**b**) Casting solution composition 15 wt % PVDF, 5 wt % PVP, 15 wt % PEG, 65 wt % TEP; (**c**) Casting solution composition 15 wt % PVDF, 5 wt % PVP, 20 wt % PEG, 60 wt % TEP.

The changes in membrane structure as a function of additives concentration in the dope solution and the exposure time during the VIPS-NIPS process was investigated. Figure 2 shows the morphology of the top surface and the cross section of the membranes prepared with different casting solutions. The nascent membranes were exposed to relative humidity (25 °C, 55% RH) over a planned time ranging from 0 to 7.5 min, and then passed in a water bath. To clearly observe the morphologies formed by NIPS and VIPS-NIPS, the upper part of the cross section was further magnified as shown in Figure 2. SEM images showed noticeable differences in the structure of the membranes produced in different operational conditions. For M1, prepared with 10 wt % PEG in the dope solution via NIPS, macrovoids were detected in the cross section. For the analogue M5 membrane, obtained with 15 wt % PEG, macrovoid formation remained, but they became shorter, and completely disappeared in M9, cast with a dope solution containing the highest investigated PEG concentration (20 wt %). For the three membranes fabricated via NIPS, SEM images denoted a dense top surface. This asymmetric structure, composed of a dense skin layer and a porous substructure, is usually formed when the concentration of polymer in the top layer remains high during the exchange of the solvent and non-solvent. In fact, phase inversion is a process strongly influenced by the casting solutions composition, which affects the solvent–non-solvent exchange rate. As largely described in literature [28,29,30], for systems with a fast phase inversion, the formation of macrovoids with finger-like structures is observed, whereas, for a slower phase inversion rate, a sponge-like matrix is preferred. Macrovoids and spongy structures can also coexist in an asymmetric membrane. Sponge-like or spherulitic membranes are commonly observed when the demixing is delayed [31,32], while if the outflow solvent diffusion rate is slower than that of non-solvent diffusion rate into the polymer-poor phase, macrovoids formation is favored [33]. The increasing of PEG concentration contributed to the increase of polymeric solution viscosity, which tended to delay TEP-water exchange, thus promoting the formation of spongy matrix and more porous top surface.

By combining VIPS with NIPS, the forming films underwent a change from the asymmetric NIPS morphology, consisting of a dense skin layer surrounded by finger-like macrovoids. When the forming membrane was in contact with water vapour for a fixed period of time prior to the immersion in the water bath, the non-solvent vapors gradually interacted with the cast dope solution. Peng et al. [34] reported that, during the VIPS process, keeping RH constant, the water chemical potential did not change, that is, the water adsorption rate was comparable for all of the nascent films. The phase separation process thus is mostly influenced by the vapour-induced time, which determined the content of adsorbed non-solvent. The symmetric porous structure observed for the membrane prepared in this work provided evidence that the water content entrapped in the film matrix was enough to induce phase separation uniformly across the whole membrane thickness. When the exposure time was fixed at 2.5 min, the morphology of all the prepared membranes became symmetric and spongy, and the pores were interconnected and formed irregular channels coexisting with the polymer network. As reported by Liu et al. [14], this bicontinuous structure is commonly observed for commercially available MF PVDF membranes (Millipore Corp., Boston, MA, USA).

No pronounced change was noticed in the membrane cross-section matrix when the VIPS time passed from 2.5 min to 7.5 min (Figure 2): for all the membranes produced through VIPS-NIPS bicontinuous structures across the membrane, a cross-section was detected.

SEM pictures of the top surface provided evidence of a uniform, highly open upper surface structure. During the VIPS step, the vapour slowly penetrated into the wet film, inducing the phase separation due to the polymer-lean and the polymer-rich phases in the forming membrane. Both nucleation and growth of the polymer-lean phase occurred during membrane formation [35]. As reported in literature, for relatively low exposure times to water vapour (generally below 30 min), nucleation represents the main mechanism and dominates over growth. Consequently, the membrane morphology becomes highly porous with open pores, i.e., the formed structure is bicontinuous.

The formation of open polymeric surface in which pores are interconnected should represent an important parameter for a potential application of the produced PVDF membranes in MF, especially considering that the bicontinuous morphology is not the simplest to obtain, so that other structures, including cellular- or nodular-like, are more prevalent [35,36,37].

The use of TEP as a solvent instead of traditional toxic solvents, such as NMP, contributed to the formation of more porous structure [17,18,23]. In fact, although thermodynamic parameters expressed in terms of polymer–solvent distance (Table 2) highlight good affinity between TEP and PVDF, molecular simulation investigations reported by Chang et al. [11] provided evidence that PVDF polymer chains were more curled in TEP in comparison to other commonly used toxic solvents, such as NMP, which may be ascribed to the higher TEP steric encumbrance and its slightly weaker interactions with polymer chains.

Kinetic studies of phase inversion behavior were demonstrated, as water passed much slower in the TEP solution than in the case of NMP [11], hence leading to a lower solvent–non-solvent exchange rate during phase separation. Moreover, a TEP-PVDF solution presented higher viscosity that limited the water inflow during phase inversion, thus promoting the formation of porous symmetric membranes.

It is generally recognized that an increase in solution viscosity strongly limits water passage through the polymer chains, thus disfavoring the formation of macrovoids in the membrane. The presence of PVP and PEG additives in the dope solutions also contributed to the formation of membranes with bicontinuous structure, merely due to the additional increase in solution viscosity. Specifically, casting solution viscosity increased in the following order: 850 cP (10 wt % PEG) > 1350 cP (15 wt % PEG) > 1600 cP (20 wt % PEG). Similar results were observed by other authors [38,39].

### 3.2. Membrane Thickness, Porosity and Contact Angle

Membrane properties dictate the performance during separation processes in water treatment. Table 4 reports thickness, porosity and contact angle of the prepared membranes. For M1, M5 and M9 prepared via NIPS, the thickness was higher than that of the analogue membranes produced by combining VIPS and NIPS at different exposure time to controlled humidity and temperature. Specifically, M1 exhibited a thickness of ~0.160 mm, which slightly increased up to ~0.164 mm for M5 and M9, mainly due to the increase of casting solution viscosity and, consequently, to the decrease in the demixing rate during phase inversion. For M1–M4, M6–M8 and M10–M12 membranes, the thickness reduced concomitantly with the increase in VIPS time of the originating film to humid air. Moreover, by fixing the operational conditions, membranes became thicker by increase PEG content in the dope solution. The observed trend may be related to the kinetic of solvent–non-solvent exchange, or rather, to the increase in the mass transfer occurring during water intake and solvent extraction.

A similar behavior was observed by Wang et al. [40], who studied the effect of PEG with different molecular weight on the PVDF hollow fibers formation. The authors reported that, for high molecular weight, PEG moved out with higher difficulty during phase separation, due to the larger molecular dimension and, consequently, lower solubility.

Membrane porosity varied between 83 and 86% for the membrane obtained via immersion–precipitation, in accordance with the PEG concentration in the polymeric solution. These values are higher than those observed for the membranes prepared through VIPS-NIPS procedure, and this behavior may be attributed to the presence of macrovoids, thus to a higher membrane specific area [41,42]. According to studies reported in literature, the bulk porosity increased with the increase of time interval during which nascent membranes were exposed to water vapour [39,43].

PEG acts as a hydrophilic absorbent agent during the VIPS step, being able to absorb water molecules in vapour form, and, at the same time, it plays a role of a former pore during the NIPS process, by leading to highly porous membranes. Similar results were reported by Tan et al. [44].

It is widely recognized that the VIPS technique promotes the formation of highly hydrophobic PVDF membranes, due to the membrane surface roughness, which leads, in some cases, even to superhydrophobic surface [34,45,46].

Peng et al. [46] described the preparation of porous and highly hydrophobic surface PVDF membranes by the gelation of a PVDF/DMA solution in open air instead of its coagulation into a water bath. The authors reported that the fabricated membranes showed micro- and nanoscale hierarchical surface roughness, which was responsible for the high contact angle (150°). In a subsequent work, Peng et al. [39] detected that a membrane water contact angle on the top and the bottom surface increased by increasing the exposure time intervals, reaching a maximum of 127.5°.

A similar trend was obtained in this study and, in accordance with SEM observations, may be attributed to the membrane roughness. Specifically, by preparing the membranes via NIPS, the contact angle of the top surface varied in the range between ~77° and ~84° for the top surface, and ~98° and ~106° for the bottom side. When VIPS was coupled with NIPS, higher contact angle values were found, reaching in the case of M12, the maximum value of 102° and 106°, for the top and the bottom surfaces, respectively.

### 3.3. Membrane Pore Size

**Figure 3:**
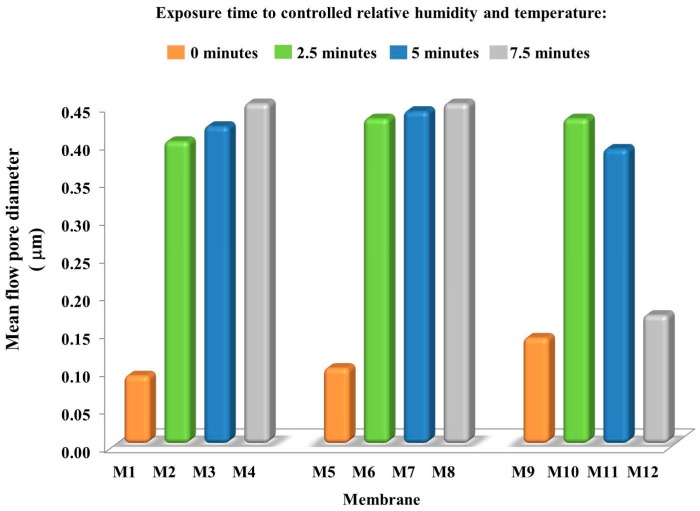
Mean flow pore diameter of the prepared membranes (in all cases, the relative standard error was less than 5%).

The pore dimension detected for the prepared membranes is reported in Figure 3. Significant differences were observed depending on the membrane fabrication method. When NIPS was applied, M1, M5 and M9 exhibited a pore dimension in the UF–MF range, i.e., ~0.09, ~0.10 and ~0.14 μm, respectively. The pore size for these three membranes increased with the increase of PEG content in the casting solution, due to its hydrophilic character and the related ability to promote pore formation. By coupling VIPS with NIPS, the scenario radically changed. In fact, all the prepared membranes showed a higher pore size, varying from 0.15 to 0.45 μm (Figure 3).

It is generally accepted that VIPS is an efficient technique to generate highly porous MF PVDF membranes [47,48]. This is strictly correlated to the intrinsic VIPS mechanism, mainly based on the use of a non-solvent in vapour form used to induce low polymer precipitation during time. When a low volatility solvent, as TEP in this case, is used to dissolve the polymer, solvent evaporation is slower than water intake, thus resulting in a phase with low polymer concentration near the film surface [12]. The lower PVDF content close to the membrane-forming surface explicates why VIPS may be considered a valid procedure to prepare membranes with highly porous surface. In addition, water as a non-solvent penetrates in the cast film with a considerable slower rate than in the case of NIPS procedure because the use of non-solvent in vapour form causes a gas phase mass transfer resistance to the membrane formation system [12]. These observations are further confirmed by looking at the mean flow pore diameter registered for the membrane cast at different delayed time. In this case, in fact, membrane pore size slightly increased when intervals time passed from 2.5 to 7.5 min (Figure 3). However, for M10–M12 membranes, prepared by adding 20 wt % PEG in the dope solution, a different trend was noted. M10 had a pore dimension of ~0.43 μm, which reduced to ~0.39 μm for M11 and further decreased to ~0.15 μm for M12. This could be related with the increase in viscosity of polymeric solution, which delays the pore formation process, contrasting with the interconnectivity of pores. The changes in membrane structure may be responsible for PVDF chain connection with the formation of a more compact matrix with a smaller pore size.

### 3.4. Membrane Pure Water Permeability (PWP)

**Figure 4:**
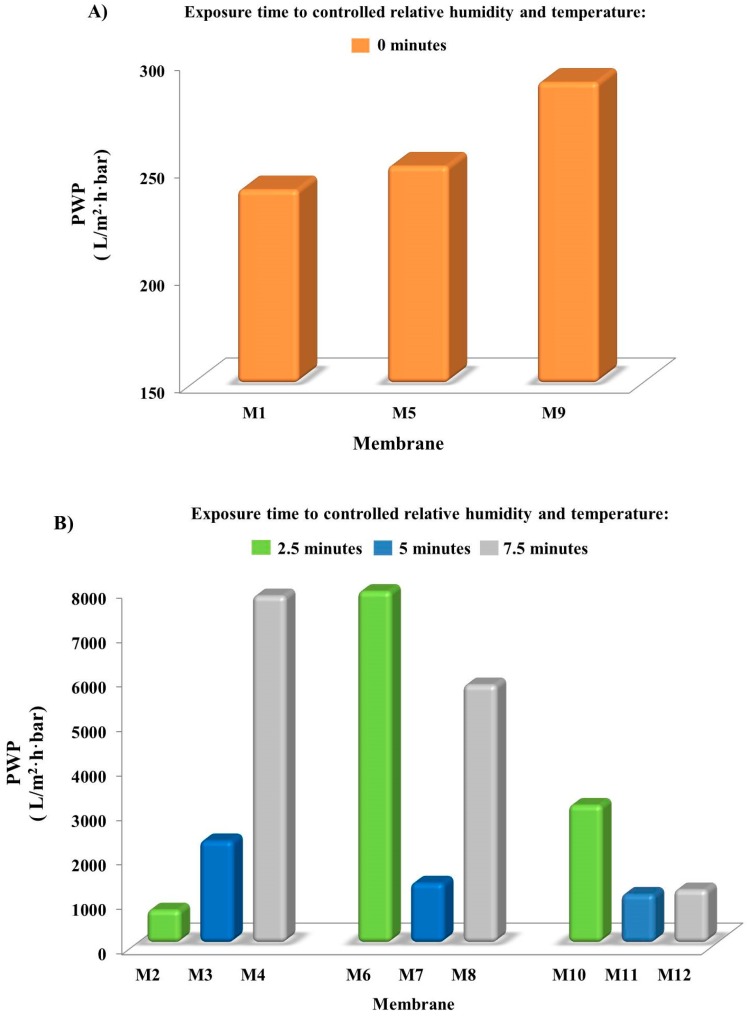
PWP of the membranes prepared via: (**A**) NIPS; (**B**) VIPS-NIPS (in all cases, the relative standard error was less than 5%).

PWP of the prepared PVDF membranes is illustrated in Figure 4. For a better observation of the results, PWP of M1, M5 and M9, produced via NIPS, are reported in a graph (Figure 4A) and that of M2–M4, M6–M8, M10–M12 obtained via VIPS-NIPS, in another graph (Figure 4B).

The direct immersion in the coagulation bath led to membranes with a PWP comprised in the range between 250 and 290 L/m^2^·h·bar. These values reflected the morphology and the pore dimension, typical of UF membranes. Even if M9 had a stronger hydrophobic character than the other two membranes prepared by immersion–precipitation that could contrast water permeability across the membrane, the higher pore size (~0.14 μm) compared to that of M1 and M5 (~0.10 μm), together with the higher porosity, allowed higher PWP.

By coupling VIPS with NIPS, PWP increases at least ten times, reaching a maximum value of 7900 L/m^2^·h·bar for M6, prepared by casting the solution containing 15 wt % PEG and exposed to water vapour for 2.5 min before the precipitation in the coagulation medium. The slow penetration of humid air inside the forming membranes for different time intervals strongly affected membrane permeability to water; that is, PWP increased with an increase in the exposure time. This is in accordance with what was reported in literature [12,26,36]. A similar effect was observed by changing the relative humidity in the environment at fixed exposure times [49]. The PWP data obtained in this work reflect membrane morphologies observed by SEM and previously discussed.

The casting solution composition also played a relevant role during membrane formation. Both PVP and PEG, used as additives, are hygroscopic agents, thus they absorb water vapour during the VIPS step, and support the formation of pores during the coagulation in the water bath. Figure 4A was highlighted by exposing the nascent films to non-solvent vapour for 2.5 min; PWP firstly increased with the increase of PEG, and then decreased. Obviously, PWP was affected by several parameters, such as thickness, porosity, contact angle and pore dimension. The combination of such factors induced the increase in PWP when PEG was increased from 10 (M2) to 15 wt % (M6), due to a comparable thickness, a higher porosity and a slight increase in mean flow pore diameter of M6.

A further increase of PEG in the dope solution up to 20 wt % was conducted to a lower PWP of M10 membrane, which showed comparable pore size of M6. Hence, the reduction in PWP was principally related to the increase in thickness and contact angle, which contrasted the water passage through the membrane, rather than pore diameter.

When the exposure time to humidity reached 5 min, PWP reduced in the following order: ~2300 L/m^2^·h·bar for M3 > ~1300 L/m^2^·h·bar for M7 ˃ 1100 L/m^2^·h·bar for M11. These results reflected the increase in membrane thickness and hydrophobicity. Promising results were registered for M4, that, together with M6 membrane, showed the highest PWP (~79,800 and ~7900 L/m^2^·h·bar, respectively). The increase of PEG concentration in the dope solution altered PWP when the nascent films were posed to controlled humid air and temperature for 7.5 min (M4, M8 and M12 in Figure 4B). Membrane thickness, total porosity, mean pore diameter and water contact angle were related to the PWP. Low thickness and contact angle, and high total porosity and large mean pore diameter contributed to increasing PWP [39].

Thus, the combination of these membrane characteristics resulted in membranes having different PWP.

In Table 5, a comparison between the obtained results and those reported in similar works is presented. Although the operational conditions, such as the RH% and the delayed time during the VIPS step, are not the same in the cited works, Peng et al. [34] and Almarzooqi et al. [36] detected a pore size below or close to 0.1 μm and tested the produced membranes in membrane distillation (MD). Fan et al. [50] described the preparation of PVDF membrane by using a double layer casting process. A first solution, composed of PVDF, LiCl and DMA, was cast to form the support layer, and a second one, containing PVDF and 1,2-propylene glycol dissolved in DMA, constituted the upper layer. By exposing the cast films to a 100% RH, it was possible to fabricated membranes with a pore size comprised in the 0.34 and 1.02 μm range, which were tested for MD. Wang et al. [51] investigated the effect of the solvent type on PVDF MF membrane formation. The research included some toxic organic solvents (DMF and DMA) and less/non-toxic solvents, such as TEP and dimethyl sulfoxide (DMSO). RH was set in this study at 30 ± 5% and the nascent membranes were exposed to water vapor for 30 s prior to the coagulation in the precipitation medium. The obtained membranes exhibited in all the cases a PWP lower than that reported in this study, even when TEP was employed as solvent.

## 4. Conclusions

Triethyl phosphate was used as a non-toxic solvent for the replacement of the commonly employed, highly toxic DMF, DMA and NMP for producing PVDF porous membranes. The effect of two different membrane preparation procedures, i.e., NIPS and VIPS-NIPS, as well as PEG concentration, was investigated. The obtained results provided evidence as through immersion–precipitation, membranes with an asymmetric structure and pore size in the range of UF, can be obtained when low PEG concentration was used (10 wt %). The increase of the pore former agent content up to 15 and 20 wt % caused a gradual change in the membrane structure, which became completely symmetric and sponge-like, with the highest employed PEG concentration. These differences reflected also the pore size and the PWP increase up until 1 μm and 290 L/m^2^·h·bar, respectively. By coupling VIPS with NIPS, membranes with a symmetric, bicontinuous morphology were fabricated. Humidity exposure time was the major affecting parameter during membrane formation. By increasing the time intervals from 2.5 to 7.5 min, it was possible to prepare membranes with different pore size, in the MF range, comprised between 0.17 and 0.45 μm, and with different PWP (from ~1100 L/m^2^·h·bar to ~7900 L/m^2^·h·bar). Thickness, porosity and water contact angle were also affected by the membrane preparation techniques. In particular, by increasing the exposure time to non-solvent in vapour form during the VIPS step, thicker and more hydrophobic membranes with higher porosity were formed. The best results were registered for the membrane prepared by casting a solution containing 15 wt % PEG, and exposed to humidity for 2.5 min prior to the coagulation in the water bath. For this membrane, the pore size was 0.43 μm, PWP ~7900 L/m^2^·h·bar, and the average porosity was ~82.8%. The results discussed above demonstrated that, by employing TEP as non-toxic solvent, PVDF membranes, with bicontinuous structure, potentially suitable for MF applications, can be prepared by adjusting the time intervals during VIPS and the casting solution composition.

## Figures and Tables

**Table 1:** Hazard statements for DMF, DMA and NMP according to Regulation (EC) No 1272/2008 [3,4].

Solvent	Hazard Statements (Classification according to Regulation (EC) No 1272/2008)	
DMF	**H226** Flammable liquid and vapour.	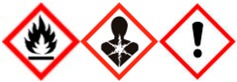
	**H312 + H332** Harmful in contact with skin or if inhaled.	
	**H319** Causes serious eye irritation.	
	**H360D** May damage the unborn child.	
	GERM CELL MUTAGENICITY:MOUSE, LYMPHOCYTE. MUTATION IN MAMMALIAN SOMATIC CELLS.	
DMA	**H312 + H332** Harmful in contact with skin or if inhaled.	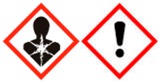
	**H319** Causes serious eye irritation.	
	**H360D** May damage the unborn child.	
	MAY CAUSE CONGENITAL MALFORMATION IN THE FETUS. PRESUMED HUMAN REPRODUCTIVE TOXICANT OVEREXPOSURE MAY CAUSE REPRODUCTIVE DISORDER(S) BASED ON TESTS WITH LABORATORY ANIMALS.	
NMP	**H315** Causes skin irritation.	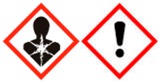
	**H319** Causes serious eye irritation.	
	**H335** May cause respiratory irritation.	
	**H360D** May damage the unborn child.	
	DAMAGE TO FETUS POSSIBLE.	
TEP	**H302** Harmful if swallowed.	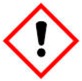
	**H319** Causes serious eye irritation.	
	THIS SUBSTANCE/MIXTURE CONTAINS NO COMPONENTS CONSIDERED TO BE EITHER PERSISTENT, BIOACCUMULATIVE AND TOXIC, OR VERY PERSISTENT AND VERY BIOACCUMULATIVE AT LEVELS OF 0.1% OR HIGHER.	

**Table 2:** Hansen solubility parameters and polymer–solvent distance.

Compound	Hydrogen Bond Force	Dispersion Force	Polar Force	Solubility Parameter	Polymer–Solvent (S) Affinity	Solvent–Non-Solvent (NS) Affinity	Reference
	δ_h_	δ_d_	δ_p_	δ_sp_	δPVDF−S	δS−NS	δ_h_
PVDF	9.2	17.2	12.5	-	-	-	[21]
TEP	9.2	16.8	11.5	22.2	1.1	33.4	[21]
DMF	11.3	17.4	13.7	24.8	2.4	31.1	[22]
DMA	11.8	17.8	14.1	22.7	1.4	32.4	[22]
NMP	7.2	18.4	12.3	22.9	2.2	35.4	[22]
WATER	42.3	15.6	16.0	47.8	-	-	[22]

**Table 3:** PVDF membranes produced by varying casting solution composition and exposure time to fixed humidity and temperature.

Membrane Code	PVDF/wt %	PVP/wt %	PEG/wt %	TEP/wt %	Exposure Time to Rh/min
M1	15	5	10	70	0
M2	15	5	10	70	2.5
M3	15	5	10	70	5
M4	15	5	10	70	7.5
M5	15	5	15	65	0
M6	15	5	15	65	2.5
M7	15	5	15	65	5
M8	15	5	15	65	7.5
M9	15	5	20	60	0
M10	15	5	20	60	2.5
M11	15	5	20	60	5
M12	15	5	20	60	7.5

**Table 4:** Thickness, porosity and contact angle of the PVDF membranes prepared by NIPS and NIPS-VIPS.

Membrane Code	Thickness (mm)	Porosity (%)	Contact Angle	
			Air Side (°)	Glass Side (°)
M1	0.160 ± 0.002	83.3 ± 0.4	77 ± 2	98 ± 2
M2	0.150 ± 0.001	80.6 ± 0.5	83 ± 1	99 ± 2
M3	0.154 ± 0.001	81.8 ± 0.6	87 ± 2	99 ± 1
M4	0.158 ± 0.001	82.4 ± 0.4	88 ± 2	100 ± 2
M5	0.164 ± 0.004	85.5 ± 0.4	78 ± 2	99 ± 2
M6	0.152 ± 0.001	82.8 ± 0.5	87 ± 2	100 ± 1
M7	0.156 ± 0.003	84.5 ± 0.4	92 ± 2	101 ± 2
M8	0.159 ± 0.001	84.9 ± 0.6	94 ± 3	104 ± 1
M9	0.164 ± 0.002	86.5 ± 0.4	84 ± 2	99 ± 1
M10	0.158 ± 0.001	85.6 ± 0.6	98 ± 2	101 ± 2
M11	0.160 ± 0.002	86.4 ± 0.5	101 ± 1	103 ± 2
M12	0.162 ± 0.002	86.6 ± 0.4	102 ± 2	106 ± 1

**Table 5:** Comparison between the most relevant results obtained in this work and those reported in literature related to PVDF membrane preparation via NIPS-VIPS.

Solvent Type	RH%	Exposure Time to Humid Air	Mean Pore Diameter	PWP	Potential Applications	Ref.
		Min	mm	L/m^2^·h·BAR		
DMA	100	0	0.11	-	Vacuum membrane distillation (VMD)	[34]
		3	0.11			
		5	0.11			
		6	0.11			
DMA	60	2	0.06	-	Direct contact membrane distillation (DCMD)	[36]
		5	0.06			
		10	0.14			
	80	2	0.07			
		5	0.13			
		10	0.14			
DMA	100	0	0.34	-	VMD	[50]
		1	0.62			
		2	0.8			
		5	1.02			
DMF	30 ± 5	0.5	-	99.6	MF	[51]
DMA				87.7		
TEP				89.1		
DMSO				272.1		
TEP	55	0	0.14	290	UF–MF	This work
		2.5	0.43	7900		
		5	0.42	2300		
		7.5	0.45	7800		
